# Laboratory and Medicine 2.1: Care for What You Wish for

**DOI:** 10.1111/ijlh.70102

**Published:** 2026-04-02

**Authors:** Marcel Levi

**Affiliations:** ^1^ Department of Vascular Medicine Amsterdam University Medical Center (Location Academic Medical Center), University of Amsterdam Amsterdam the Netherlands

## Abstract

Over the past few decades, healthcare has made remarkable advances. Across nearly all fields, diseases are better understood, and diagnostic and therapeutic options have expanded dramatically. Laboratory medicine is also developing at a rapid pace and is playing an increasingly central role in the diagnostic process, as well as in the monitoring of therapy and disease progression. For many conditions—including cardiovascular disease and cancer—patient outcomes have improved spectacularly. However, this success has also created new challenges. Death from acute illness has increasingly been replaced by survival with chronic disease. An aging—though not necessarily healthy—population demands substantial personal, financial, and organizational support. Hyperspecialization is poorly suited to meeting these complex needs. Moreover, efficiency‐driven centralization of care, encompassing both clinical services and laboratories, may be necessary but often introduces new problems that can only be addressed through innovation and technology. Most importantly, strong professional leadership is essential. With healthcare professionals in the lead, we can ensure a resilient and sustainable healthcare system, supported by high‐quality laboratory services, in which complex issues such as superspecialization, centralization, and efficiency are addressed in an optimal and integrated manner.

## 1 Modern Medicine

It was the renowned North American physician William Osler who—impressed by the rapid advances in medical knowledge and understanding at the turn of the twentieth century—declared: “… Never has the outlook for the profession been brighter. Everywhere the physician is better trained and better equipped than he was 25 years ago. Disease is understood more thoroughly, studied more carefully, and treated more skillfully…” [1]. Indeed, during or shortly after Osler's era, hormones and vitamins were discovered, electrocardiography was introduced, blood groups were identified, vaccination began to take hold, and the medical application of Röntgen rays was actively explored and slowly introduced into clinical medicine.

It can be argued, however, that 100 years later the expansion of biomedical understanding—translated into improved diagnostic tools and more effective therapeutic strategies for numerous diseases—is even more impressive. Indeed, never before has the growth in biomedical knowledge been so steep, and there are no indications that this rapid escalation is approaching an end. Arguably, we are living in a golden age of medicine.

**FIGURE 1:**
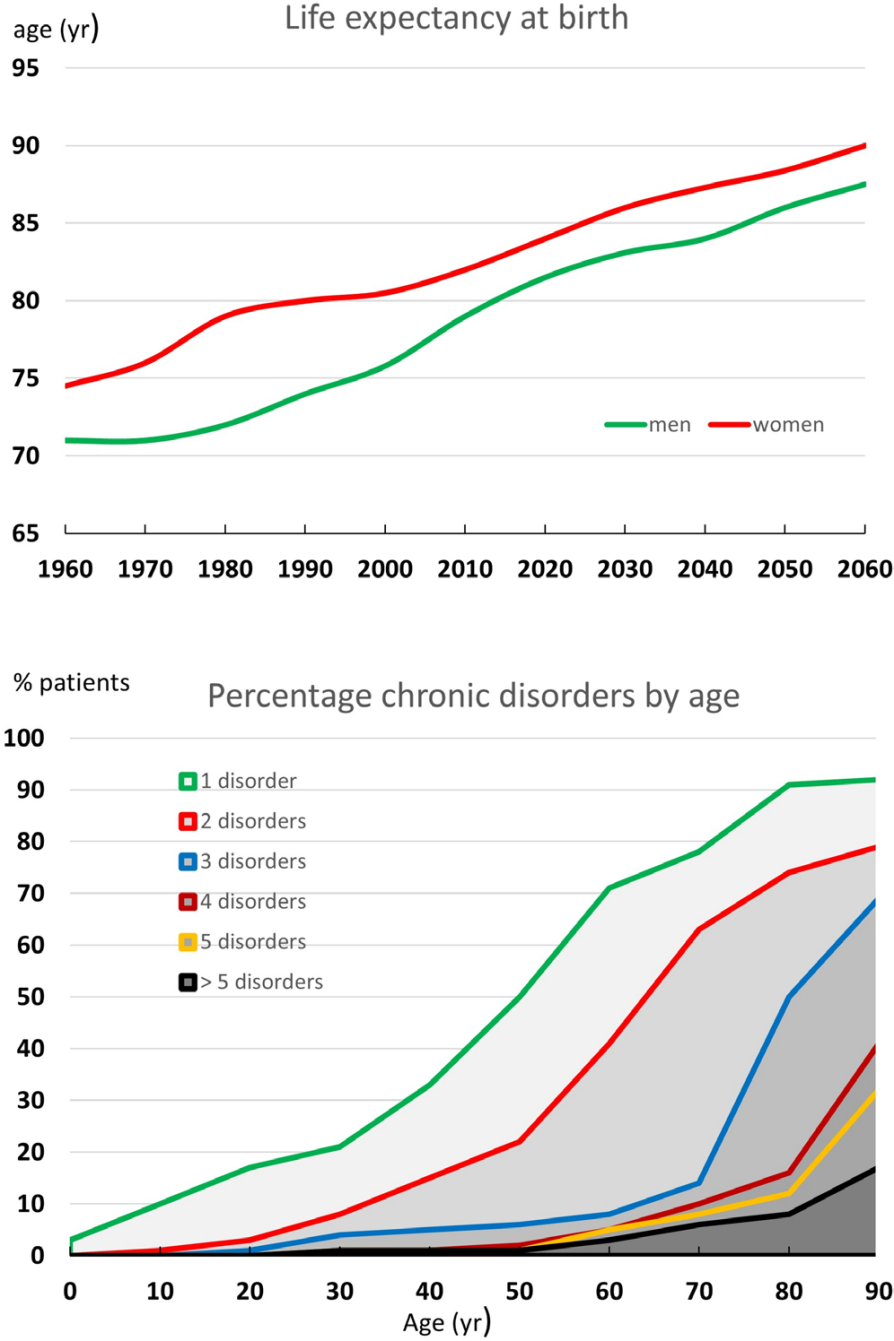
Upper panel: Development of life expectancy at birth over recent decades. The numbers for 2025 and further are predictions. Lower panel: Percentage of patients with 1 to > 5 chronic disorders at various age intervals. *Source:* European Bureau of Statistics (2024); RIVM, the Netherlands (2022).

For the first time in history, medicine has made a substantial contribution to increased longevity. While the most substantial rise in life expectancy in the 19th and early 20th century can largely be attributed to improved social conditions, better public hygiene, and reduced warfare, the sharp increase in survival in the last decades is predominantly due to more effective treatment of life‐threatening diseases. In the Western world, mortality from cardiovascular disease has been reduced by approximately 50% and has been overtaken by cancer as the leading cause of death [2]. Importantly, cancer‐related mortality is also gradually declining. Overall, life expectancy is increasing rapidly worldwide due to better healthcare and it has been estimated by the World Population Prospects of the United Nations that a child born in the Western World today has a 50% chance of reaching the age of 100 years (Figure 1).

Regrettably, survival following acute, severe illness often results in an increased burden of chronic disease, and many of the years gained in life expectancy are exchanged for years lived with impaired quality of life. Consequently, chronic multimorbidity in elderly individuals has emerged as a major challenge for modern medicine. Nevertheless, the expansion of biomedical knowledge and therapeutic capabilities has not only prolonged life. Perhaps even more remarkable is the transformative impact of modern management strategies on previously debilitating conditions, such as rheumatoid arthritis, chronic infectious hepatitis, inborn errors, and congenital heart disease.

## 2 Crisis, What Crisis?

**FIGURE 2:**
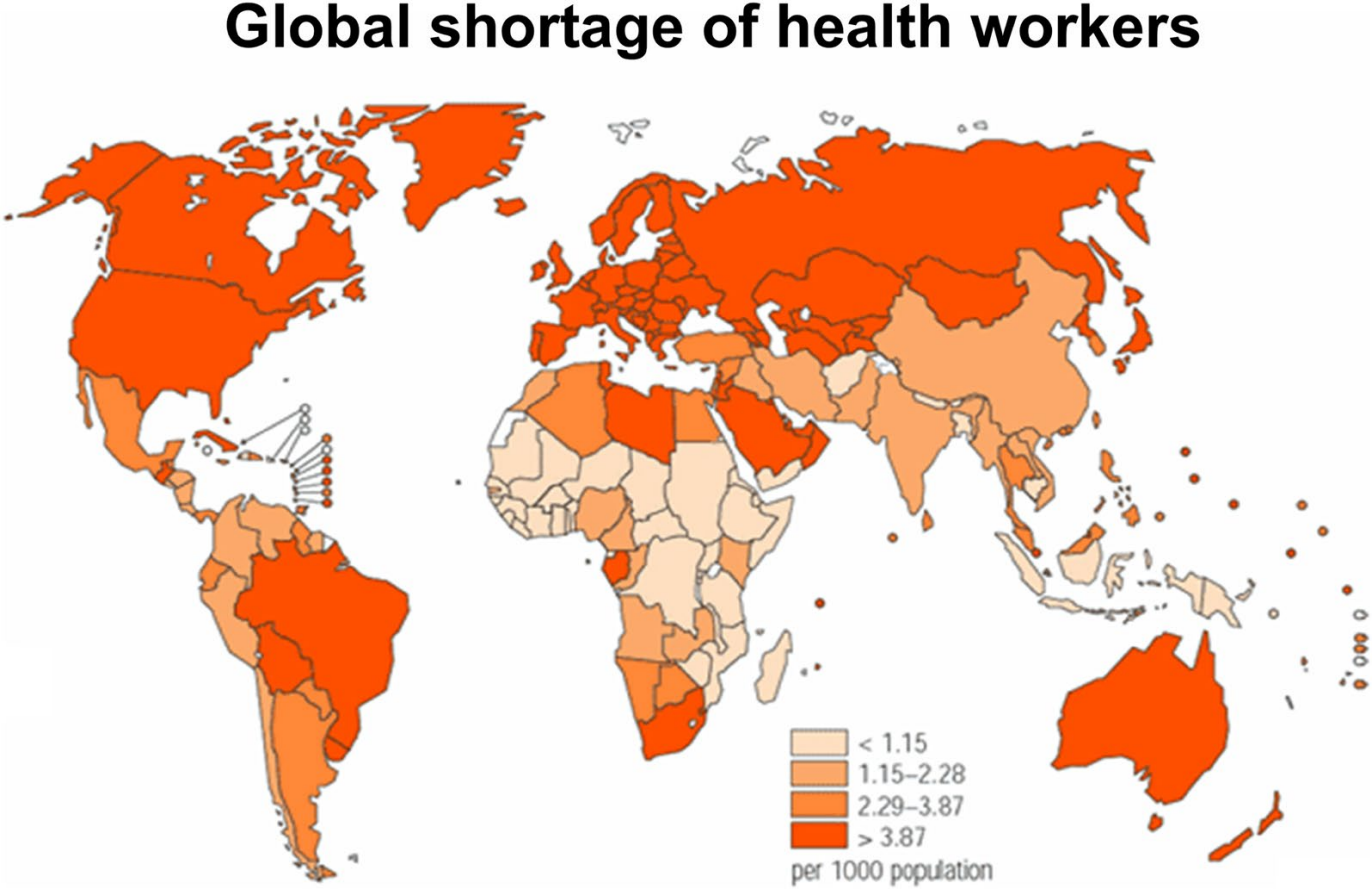
Shortages of health care workers worldwide expressed as number per 1000 population. Data are derived from national reporting in the Global Health Workforce Statistics database. *Source:* World Health Organization (2024).

Nevertheless, an increasing number of articles in both medical journals and the lay press describe an imminent—or already unfolding—healthcare crisis. According to these reports, healthcare systems worldwide are facing staff shortages (likely due to a shrinking workforce and increasing demand in an elderly population, most pronounced in affluent countries) (Figure 2), escalating costs that appear to be spiraling out of control, insufficient capacity resulting in limited access and unacceptably long waiting lists, and increasingly complex logistics. While these problems undeniably exist and pose serious threats to the delivery of appropriate healthcare, one could argue that they are, at least in part, a consequence of our own success.

Medicine and healthcare have become so effective, and the range of available diagnostic and therapeutic options has expanded so dramatically, that the balance between demand for medical care and the supply of services and resources required to deliver it is becoming increasingly strained. This imbalance inevitably creates friction. However, it may also be interpreted as a temporary phenomenon associated with a transitional phase in healthcare, one that will be alleviated by further scientific insights, technological advances, and innovative models of care delivery.

History offers several examples in which predicted labor shortages were thought to impede progress, only to be overcome by technological innovation. Similarly, rising healthcare costs are largely driven by improved treatments—such as advanced interventions and novel pharmaceuticals—and this, arguably, is precisely what society has long demanded. Population aging will undoubtedly increase healthcare expenditures; however, this concern may be somewhat overstated. As individuals reach older ages in better overall health, the potential workforce available to provide care and support also increases. Indeed, if one conservatively assumes that individuals require substantial medical care and support during the final 15 years of life, it can be calculated that the proportion of people living within this final 15‐year period relative to those who are not is, in fact, decreasing [3].

## 3 Medicine in the 21st Century and Health Care Professionals

The question is how we as health care professionals are responding to this biomedical revolution that we are dealing with. In some situations medicine gets increasingly complex, requiring highly specific and multifaceted infrastructure and demanding skills of medical and paramedical professionals. One of the responses of the medical professionals is subspecialization [4]. Subspecialization does not only occur in traditionally “broad” disciplines, such as internal medicine, surgery, or pediatrics, but is increasingly seen in virtually all medical specialisms. We now see interventional cardiologists or cardio‐electrophysiologists, fertility specialists, immuno‐dermatologists, ophthalmologists who entirely focus on the anterior chamber of the eye, and ear‐nose‐throat specialists who only want to hear about the ear ossicles. It seems that the subspecialization of all medical disciplines is evolving every year and is not going to stop for a while.

Obviously it is unavoidable that ever increasing complexity in medicine is addressed by mounting specialization by physicians. It can even be regarded as a very positive point that we have colleagues that are highly specialized and can keep up with even the slightest detail in their area, much to the advantage of their patients. However, there is also a risk associated with the absolute focus on one organ, or part of an organ or even a specific tissue within this organ part or an explicit abnormal cell type within this tissue. Subspecialization may lead to narrow vision, to treatment of a disease, diseased tissue or a diseased cell rather than treatment of a patient. It may also cause overestimation of the potential of medicine, as there will often be a remedy against even advanced disease but this may be detrimental for the organism as a whole and the overall wellbeing of a patient.

In addition, there is an increasing number of patients who have medical problems in more than one area or it is not yet clear what the diagnosis is. It has been shown that the treatment of these patients by specialists is wanting, and it lacks an integrated management of multiple problems [5]. In fact, in many cases subspecialization unfortunately does not only mean more knowledge in one specific area but also not wanting to be involved in any other area of medicine. That means that a patient with multimorbidity needs to see many different specialists and may move from one waiting room to the other while there is no single doctor that oversees the patient as a whole [6]. Interestingly, there has never been any capacity planning for all these subspecialists which has led to an overkill in highly specialized doctors and a shortage of general physicians.

The notion that medical knowledge has grown too big that it has become impossible to be overseen and covered by one single physician is often heard but never proven. It is actually rather a sign of lack of ambition and comfortably degrading one's own intellectual ability. Of course it is impossible to oversee all new developments and details in the broad field of medicine, but the scope could probably be larger than the current narrow subspecialist view. It is likely that with the ever improving organization of practical medical knowledge in evidence‐based databases that are easily accessible from portable devices it has become probably easier to cover broad areas of medicine and offer patients the right treatment. This, however, will only be possible with a sustained and wide‐ranging interest in medicine, integral attention for ill humans and the conviction that optimal medicine is patient‐oriented rather than doctor‐oriented.

## 4 Solving the Paradox: Generalism in an Ever Subspecializing Environment

How can we reconcile the ideas that modern medicine needs focus, concentration and, as a consequence, subspecialization on one hand and that patients need a general rather than a subspecialized approach on the other hand? The solution is actually rather simple. Subspecialization is good but only if it is done by physicians who are willing to think and act also beyond the borders of their specific interest. So when a patient that is treated with steroids for lymphoma develops diabetes, the hematologist must handle this complication as well rather than sending the patient to an endocrinologist for blood sugar control. And ordinary postoperative respiratory tract infections should ideally be treated by surgeons. And is there any reason why a cardiologist could not manage mild renal insufficiency? And why should an elderly patient with chronic obstructive pulmonary disease, mild heart failure and atrial fibrillation, diabetes, gallstones and a stable chronic lymphocytic leukemia consult a pulmonologist, cardiologist, endocrinologist, gastroenterologist and hematologist whereas a broadly oriented internist can cover all these conditions by himself and according to the most recent insights? So you can be subspecialized as much as you want as long as you will take care of the rest as well. Obviously, physicians need to determine the limit of their skills and knowledge and to assess individually how all‐encompassing they can be and for which circumstances it is in the patient's interest to refer him to a specialist colleague but certainly the bulk of the problems should be handled by one doctor, however subspecialized he or she may be. If we would (re‐)adopt this model of generalism in our current subspecializing environment and re‐introduce this concept in our medical curricula and training programs, our patients will be able to even more benefit from all the advances modern medicine has to offer [7].

## 5 Laboratory Medicine in Modern Health Care

Against the backdrop of a rapidly changing and evolving medical field, laboratory medicine is, of course, also subject to major changes. An increasing number of laboratory tests are becoming available to refine and improve disease diagnostics. Laboratory markers for early diagnosis are being introduced, existing tests are gaining sensitivity and specificity, and testing methods are being simplified and thus becoming more widely available. Add to this the enormous automation of laboratory diagnostics, and the modern clinical laboratory is hardly recognizable compared to what it was 20 years ago. The number of laboratory tests performed is also increasing at a rapid pace. A British study of laboratory testing conducted in primary care showed an increase from 15 to 50 laboratory tests per 10 000 people per year over the past 15 years, mainly—but not exclusively—among older individuals [8]. A similar trend was observed in hospital laboratories [9].

**FIGURE 3:**
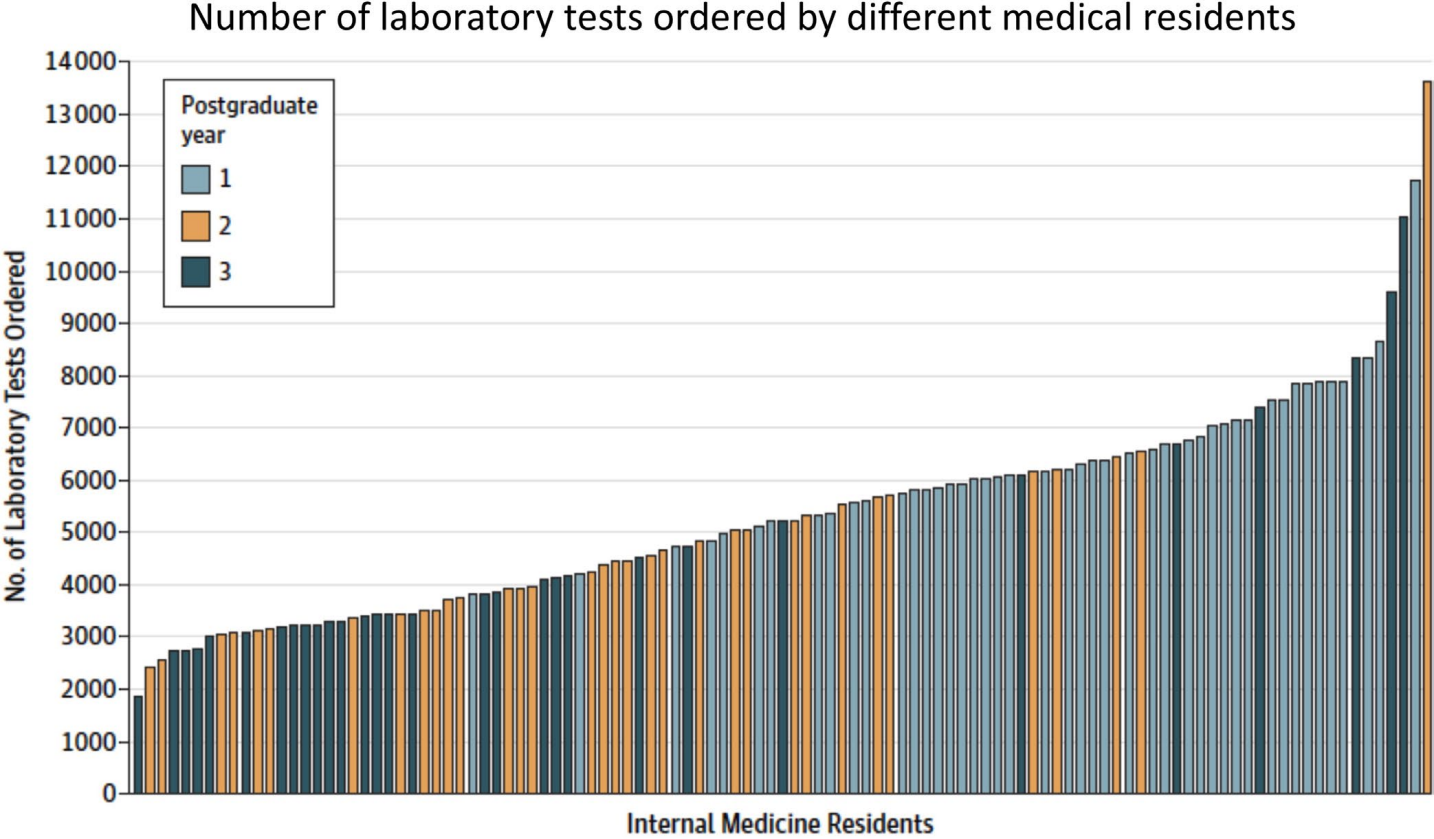
Variation in laboratory testing of cohorts medical residents on general wards of internal medicine in a teaching hospital (adapted from Geleris et al. [9]). Note the correlation between year of training and ordering habit.

The question, of course, is whether all of these tests are truly necessary. Studies showing extreme variation in practice among identical populations with the same diagnosis suggest that there may be overuse of laboratory testing in some areas (Figure 3) [10]. Consequently, the role of the laboratory consultant appears to be rapidly shifting from someone who only performs tests to someone who has a much broader task, that is, advising on the appropriate indication for testing within the diagnostic process and helping to correctly interpret laboratory results, and who acts as a consultant to other clinicians involved in patient care. This role is evolving into that of a specialist who is consulted before expensive or complex tests are ordered, or who provides feedback when a routine test is repeatedly ordered or interpreted incorrectly. This means that the laboratory specialist must increasingly leave the laboratory and be present in multidisciplinary patient case discussions, protocol and guideline committees, and elsewhere in the hospital or clinical practice. It may therefore be worth considering the inclusion of clinical rotations in the training of laboratory specialists in order to facilitate interaction between future laboratory physicians and other clinicians, despite practical difficulties to achieve this.

Another point of discussion is whether the increase in diagnostic testing is always meaningful. Very often, new diagnostic possibilities are added to the existing repertoire rather than replacing established test panels. The question arises whether, with the introduction of a new—better—test, a number of “old” tests should no longer be offered. This is particularly an issue with the introduction of molecular techniques (e.g., in microbiology or malignant hematology), which often provide improved, more refined, and faster diagnostic information, but in many cases are performed alongside existing diagnostic methods that are themselves costly and labor‐intensive. There may well be significant efficiency gains to be achieved here.

## 6 Is Centralization the Answer?

The rapid advances in molecular genetics, biotechnology, and analytic techniques, coupled with detailed insights into complex mechanisms such as immunology, host defense, coagulation, metabolism, tissue differentiation, and cell regulation, have brought about revolutionary changes in laboratory medicine in the current era. At the same time, as the complexity and costs of these technologies continue to rise, centralization of laboratory facilities has become increasingly common [11, 12]. Numerous studies across various areas of medicine have demonstrated a clear relationship between healthcare volume and clinically relevant outcomes, and there is little reason to believe that laboratory medicine would be an exception. Moreover, economic principles of scale predict substantial efficiency gains when laboratory services are centralized.

However, centralization and outsourcing are not without downsides [13]. While there is a clear rationale for outsourcing supporting services such as cleaning or catering, it can be argued that diagnostic laboratories are at the very heart of patient care, making it questionable to relocate them offsite and away from other clinicians [14] In the previous paragraph it was suggested that laboratory clinicians should get closer to clinical colleagues, and an off‐site centralized laboratory at a distance may actually hamper that step. Also, the short‐term financial benefits of an offsite central laboratory may be offset by declining quality and service levels, loss of tailored facilities, and—most critically—a complete erosion of knowledge and experience within the organization, resulting in greater dependence on external providers. Management and improvement of an underperforming laboratory service is often replaced by the (frequently unsuccessful) management of a contract. Moreover, outsourcing creates a workforce that is removed from the core mission of the institution; employees may feel marginalized, struggle to engage with the organization's values and objectives, and fail to develop a genuine sense of belonging.

Hence, it is crucial to find the best of both worlds where the benefits of centralization are combined with the need for decentralized presence and integration within clinical activities.

## 7 How We Can Realize Appropriate Change and Transformation

It is often claimed that healthcare is only limited in its ability to implement major changes, as for example recently stated by Dr. Hans Kluge, European Director of the World Health Organization. However, this assertion is not supported by the enormous transformations that have taken place over the past decades. For example, thanks to new medical insights and technologies, the need for prolonged hospital stays has been greatly reduced, resulting in a drastic decrease in the average length of stay. Another example is the, without exaggeration, dramatic shift of care from secondary to primary care: consider diabetes, chronic respiratory diseases, mental health conditions, heart failure, or musculoskeletal disorders. It is now estimated that millions of people with these conditions are treated in primary care or practice nurses [15]. How can one claim that we cannot change? These are clear illustrations that healthcare is indeed capable of significant transformation.

There are plenty of plans and ideas, but in the coming years, we really need to push to put them into practice. The question is who will implement all these changes? Recent history teaches us that managers, economists, politicians, and public administration experts are not capable of fully understanding healthcare in all its nuances, let alone keeping pace with rapidly evolving medicine. The only viable option is for healthcare professionals themselves to take the reins [16]. They are the one group that truly knows what is happening in the consultation room, the labs, emergency department, or home care. Yet they seem immersed in their work, worn down by increasing administrative burdens, absorbed in their professional literature, and tucked away in their professional organizations—literally struck dumb when it comes to taking a leading role in healthcare and presenting it externally.

Healthcare institutions are, by definition, professional organizations, and there is ample evidence that leadership by professionals has a positive effect on outcomes [17]. Professionals are better than anyone else at identifying priorities—for example, when purchasing new equipment or making other efficient investments—and can make well‐considered decisions without being swayed by fleeting trends or vague arguments. Furthermore, professionals speak a language among themselves that is not always understood by nonprofessional managers, and conversely, managerial language is often unintelligible to healthcare professionals. Terms such as governance, production, leverage, or benchmark tend to elicit a questioning look—or at least irritation—among healthcare professionals. It is a positive sign that the younger generation of medical professionals shows much more interest in the organization of the profession and does not hesitate to develop expertise in this area. There is in fact ample research showing that hospitals, research labs, and other professional environments led by professionals achieve better outcomes than those managed by other types of managers.

## 8 Conclusion

Medicine and laboratories represent a rapidly changing environment where continuous improvements lead to better outcomes for patients. However, the pace of change necessitates relatively swift adaptation of our way of working and our organizations. This is generally successful, but sometimes it takes some delay, creating capacity issues, staff shortages, and financial challenges. With professionals in the lead, however, we may ensure an increasingly robust healthcare system, supported by the necessary laboratory services, and can complex matters such as superspecialization, centralization, and efficiency be optimally addressed.

## Author Contributions

This manuscript was conceived, developed, and written by the sole author, M. Levi.

## Funding

The author has nothing to report.

## Ethics Statement

The author has nothing to report.

## Consent

The author has nothing to report.

## Conflicts of Interest

The author declares no conflicts of interest.

## Data Availability

Data sharing not applicable to this article as no datasets were generated or analysed during the current study.
